# TRPC1-STIM1 activation modulates transforming growth factor β-induced epithelial-to-mesenchymal transition

**DOI:** 10.18632/oncotarget.12895

**Published:** 2016-10-25

**Authors:** Anne Schaar, Pramod Sukumaran, Yuyang Sun, Archana Dhasarathy, Brij B Singh

**Affiliations:** ^1^ Department of Biomedical Sciences, School of Medicine and Health Sciences, University of North Dakota, Grand Forks, ND 58201, USA

**Keywords:** calcium, SOCE, TRPC1-STIM1, calpain, EMT

## Abstract

Activation of Epithelial-to-Mesenchymal Transition (EMT) is important for tumor metastasis. Although growth factors such as TGFβ and EGF have been shown to induce EMT in breast epithelial cells, the mechanism resulting in migration is not well understood. Herein, we provide evidence that Ca^2+^ entry into the cell, especially upon store-depletion, plays an important role in TGFβ-induced EMT by promoting cellular migration and potentially leading to metastasis. The increased migration by TGFβ in non-cancerous cells was due to the loss of E-cadherin along with a subsequent increase in N-cadherin levels. Importantly, TGFβ-treatment increases store-mediated Ca^2+^ entry, which was essential for the activation of calpain leading to the loss of E-cadherin and MMP activation. Inhibition of Ca^2+^ entry by using Ca^2+^ channel blocker SKF-96365, significantly decreased Ca^2+^ entry, decreased TGFβ-induced calpain activation, and suppressed the loss of E-cadherin along with inhibiting cell migration. Furthermore, TRPC1 function as an endogenous Ca^2+^ entry channel and silencing of either TRPC1 or its activator, STIM1, significantly decreased TGFβ induced Ca^2+^ entry, inhibited TGFβ-mediated calpain activation and cell migration. In contrast, overexpression of TRPC1 showed increased Ca^2+^ entry and promoted TGFβ-mediated cell migration. Moreover, increased TRPC1 expression was observed in ductal carcinoma cells. Together these results suggest that disrupting Ca^2+^ influx via TRPC1/STIM1 mechanism reduces calpain activity, which could restore intercellular junction proteins thereby inhibiting EMT induced motility.

## INTRODUCTION

Tumor metastasis is the principal cause of cancer-associated deaths and accounts for over 90% of all cancer deaths [^1, 2]^. Metastasis occurs when a malignant cell migrates from a primary organ through the blood stream or lymphatic system to a secondary site causing new tumor growth [^3–5]^. The steps of metastasis include: a) invasion of cancer cells followed by entry into systemic circulation (intravasation), b) movement from the circulatory system into a new host tissue (extravasation), and c) initiating proliferation and growth of the secondary tumor. Thus, without the ability of a tumor cell to migrate, carcinoma cells will remain at the primary site where current therapeutics can be administered, thereby decreasing the probability of patient mortality.

For metastasis to occur, a tumor cell shifts from a polarized epithelial state to an invasive migratory phenotype through a process known as Epithelial-to-Mesenchymal Transition (EMT) [^6]^. Epithelial cells form a sheet or layers of cells that are tightly connected laterally by specialized junction structures and loss of these intercellular junctions, cytoskeletal reorganization, and expression of mesenchymal proteins leads to increased migration [^7, 8]^. A known inducer of EMT is the cytokine Transforming Growth Factor Beta, TGFβ, which has been shown to induce EMT via the non-conical pathway in epithelial cells along with increasing cancer cell invasiveness [^9–11]^. During the first few hours of TGFβ exposure, the cell undergoes rapid and dynamic changes in gene regulation with morphological and migratory changes seen between 24 and 48 hours [^12, 13]^. TGFβ initiated changes proceed through a multitude of intracellular signaling pathways including Smad-dependent (canonical) and Smad-independent (non-canonical) pathways [^14]^. In addition to TGFβ, several other tyrosine kinase receptor agonists and growth factors, including FGF, IGF, EGF, and PDGF have also been shown to play a critical role in regulating EMT-like morphogenetic events. Although the role and molecular mechanisms of TGFβ and other agonists in EMT have been extensively studied, relatively little is known about the intracellular signaling pathways, especially the non-canonical pathway, that drive rapid responses to EMT-inducing signals. Thus, it could be anticipated that a common signaling molecule could be essential in regulating EMT.

TGFβ induced EMT down regulates epithelial factors, such as E-cadherin, and increases expression of mesenchymal factors. Although changes in gene expression is important for the progression of EMT, the degradation of the extracellular matrix (ECM) allowing a tumor cell to migrate is equally as important. The cell-cell adhesion molecule epithelial cadherin (E-cadherin) functions as the gatekeeper of the epithelial state and partial loss of E-cadherin has been associated with carcinoma progression and poor prognosis in various human and mouse tumors [15]. Importantly, forced expression of E-cadherin in invasive carcinoma cells have been shown to inhibit their ability to invade and metastasize, further underscoring the importance of E-cadherin loss in migration and metastasis [16, 17]. Loss of function and decreased expression of E-cadherin in EMT is countered by the increased expression of mesenchymal neural cadherin (N-cadherin) through a process known as the ‘cadherin switch’ [18]. Expression of N-cadherin and the intermediate filament vimentin by malignant cells promotes transendothelial migration, a consequence of EMT. These EMT transcriptional expression changes in epithelial and mesenchymal factors occurs concurrently to the degradation of the ECM. This degradation is assisted by the cleavage of E-cadherin in both localized and metastatic tumors and is believed to be mediated by the calcium (Ca^2+^)-dependent calpain family of cysteine proteases [19, 20]. Calpains are activated by TGFβ, possibly through the PI3K/Akt signaling pathway [21], and their activity level is correlated to tumor cell invasiveness. Over-activation of calpains triggers a reduction in the ECM and an increase in tumor cell invasiveness [22]. Further, use of calpain inhibitors such as calpeptin reduces tumor invasiveness and the quantity of E-cadherin cleavage [19]. Although the importance of calpain activity in tumor cell motility has been investigated, the ion channel and the source of Ca^2+^ entry necessary for calpain activation has not yet been identified. Another important family of proteases responsible for degradation of the ECM are the Ca^2+^ dependent matrix metalloproteinases (MMPs). TGFβ is known to increase MMP mRNA and protein levels thru p38 MAPK and ERK1/2 pathways leading to increased tumor invasiveness [^23, 24]^. Importantly, in breast cancer cells, Ca^2+^ also modulates the expression of junctional proteins. It has been shown that N-cadherin and vimentin proteins expression is reduced by intracellular Ca^2+^ chelation and partially regulated by Ca^2+^ channels [25]. Thus, identification of Ca^2+^ sources and channels involved in EMT intracellular pathways may be key to understanding the mechanisms of metastasis.

The second messenger Ca^2+^ is critical in a multitude of cellular processes including cell proliferation, motility, and cellular migration [26, 27]. Though the relationship between fluctuations in Ca^2+^ concentration and migration has been previously studied [28–30], how Ca^2+^ is involved in EMT induced cell metastasis has yet to be uncovered. Changes in intracellular Ca^2+^ are the result of either the release of Ca^2+^ stores from the endoplasmic reticulum (ER) that initiates store-operated Ca^2+^ entry (SOCE) mechanism, or direct Ca^2+^ entry from the extracellular space upon membrane depolarization; however in non-excitable cells intracellular Ca^2+^ is mainly achieved through the SOCE mechanism [31]. During initiation of SOCE (when ER Ca^2+^ stores are depleted) Stromal Interaction Molecule 1 (STIM1) is shown to aggregate to the endoplasmic reticulum-plasma membrane (ER-PM) junctions, where it interacts with proteins such as the Transient Receptor Potential Canonical 1 (TRPC1) and Orai1 channels causing Ca^2+^ influx [32]. Recent studies have suggested the EMT pathway is dependent on Ca^2+^ and there is strong evidence to suggest STIM1 and TRPCs/Orais are involved in regulating Ca^2+^ influx in various cell types [25, 28, 33, 34]. Though the consequences of EMT are well documented, the overall understanding of the intracellular signal transduction pathway and the role of Ca^2+^ channels leading to EMT that promotes tumor metastasis is largely unknown. In this study we examined the effects of disrupting Ca^2+^ influx in both non-cancerous and tumor cell lines and identified TRPC1 and STIM1 as the endogenous Ca^2+^ signaling machinery essential for EMT. Our results show that TGFβ-treatment induces calpain activity upon Ca^2+^ influx via the TRPC1 channel, which decreases its substrate E-cadherin, thereby initiating EMT induced cell motility. Further, TRPC1 mediated Ca^2+^ influx plays a role in the expression of N-cadherin and Vimentin. Finally, inhibition of TRPC1 channel or silencing of STIM1 or TRPC1 expression inhibited TGFβ-induced EMT, suggesting that these could be used as potential drug targets for inhibiting cell metastasis.

## RESULTS

### TGFβ induces EMT in normal cells

**Figure 1:**
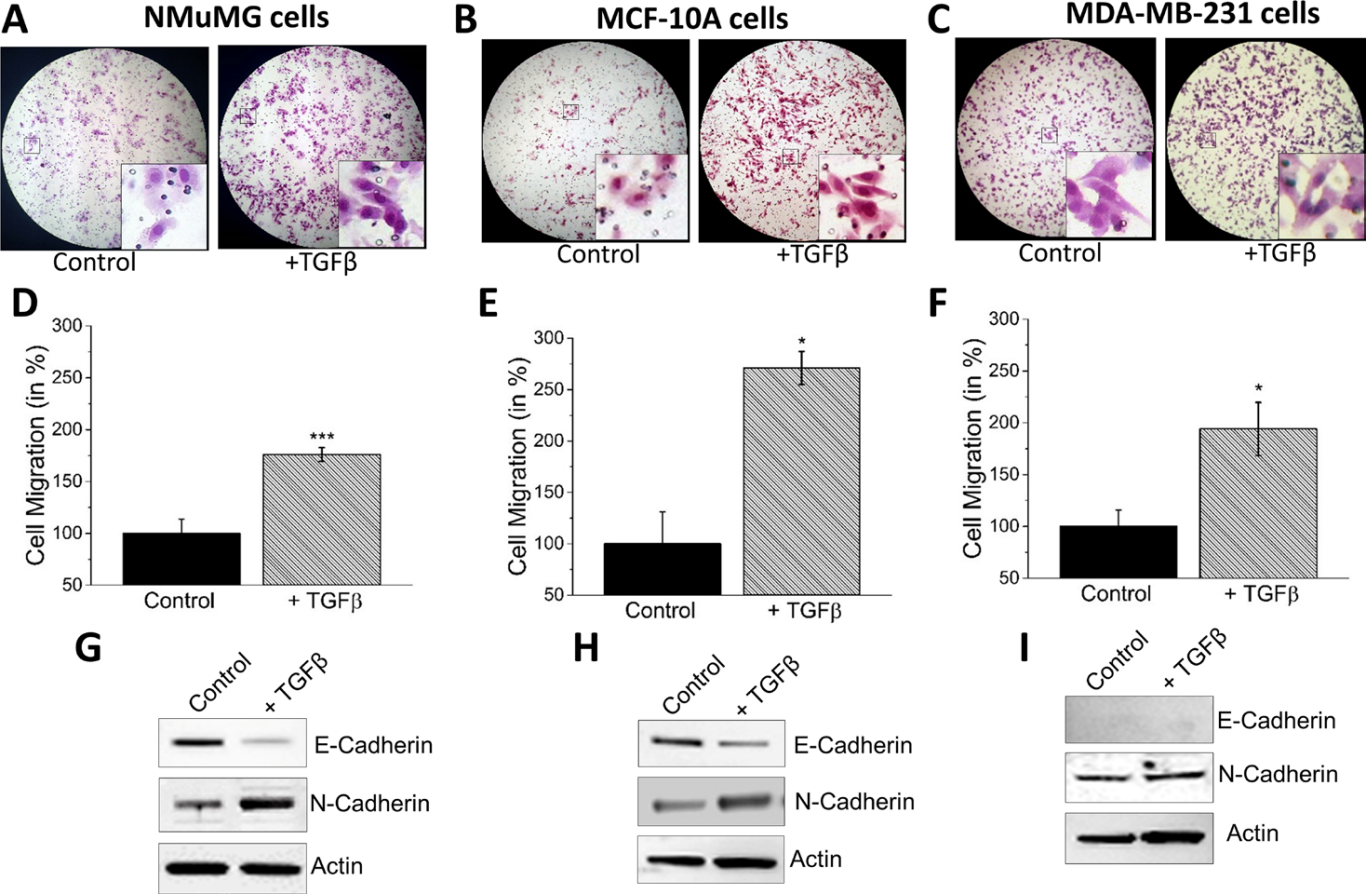
TGFβ induces EMT in NMuMG, MCF-10a and MDA-MB-231 cells NMuMG. (**A**), MCF-10a (**B**), and MDA-MB-231 (**C**) cells were treated with 10 ng/mL TGFβ for 48 hours before being trypsinized, resuspended in serum free media, placed in transwell inserts with 8 uM pore membranes, and incubated for 6 hours and 24 hours (MCF-10A). Nonmigrating cells were removed from upper membrane and migrated cells were stained with hematoxylin and fixed. Magnification at 10×. (B, E, H) Four random fields at 10× were counted indicating total cells migrated, shown as percent change in NMuMG (**D**), MCF-10a (**E**), and MDA-MB-231 (**F**). Western blots showing E-cadherin and N-cadherin protein levels normalized to actin in NMuMG (**G**), MCF-10a (**H**), and MDA-MB-231 (**I**) after 48 hour treatment of 10 ng/mL TGFβ.

Murine mammary epithelial NMuMG cells are responsive to TGFβ and induces EMT, however the mechanisms and the proteins involved are not fully understood [35]. Thus, we initially used TGFβ to induce EMT in NMuMG cells using a transwell migration assay. As indicated in Figure 1, results from the migration assay showed elongated cell morphology and increased migration as compared to the untreated control cells (Figure 1A). Quantification of the number of cells migrated indeed showed a significant increase in the migration of NMuMG cells treated with TGFβ indicating EMT induction (Figure 1D). To confirm activation of EMT, we evaluated the expression of E-cadherin and N-cadherin. E-cadherin was decreased along with a subsequent increase in the N-cadherin level (Figure 1G). To further support our results, experiments were also performed on the non-metastatic cell line MCF-10A and the metastatic cell line MDA-MB-231. Similar to NMuMG cells, the MCF-10A, a normal breast epithelial cell line, also undergoes EMT when treated with TGFβ and increased motility is observed [35]. MCF-10A cells showed the same mesenchymal morphology and increased migration upon TGFβ stimulation (Figure 1B). Furthermore, quantification of the migrated cells showed a more than two-fold increase in migration of MCF-10A cells after being pre-treated with TGFβ (Figure 1E) and a subsequent reduction of E-cadherin and an increase in N-cadherin expression was observed (Figure 1H). The human breast cancer cell line MDA-MB-231 was used as a positive control as it naturally expresses high levels of EMT markers, is innately metastatic, and increases migration when treated with TGFβ due to degradation of the ECM by MMPs [24, 35, 36]. As expected, MDA-MB-231 cells treated with TGFβ also increased migration; however, the degree of increase was less as compared with NMuMG and MCF-10A cells (Figure 1C, 1F). MDA-MB-231 cells showed no E-cadherin expression as it is an E-cadherin negative cell line and N-cadherin remained the same after TGFβ-inducement (Figure 1I). Together, these results suggest that TGFβ induces cell migration by inducing EMT in normal and metastatic breast cells.

### TGFβ increases store-mediated Ca^2+^ entry and [Ca^2+^]_i_

**Figure 2:**
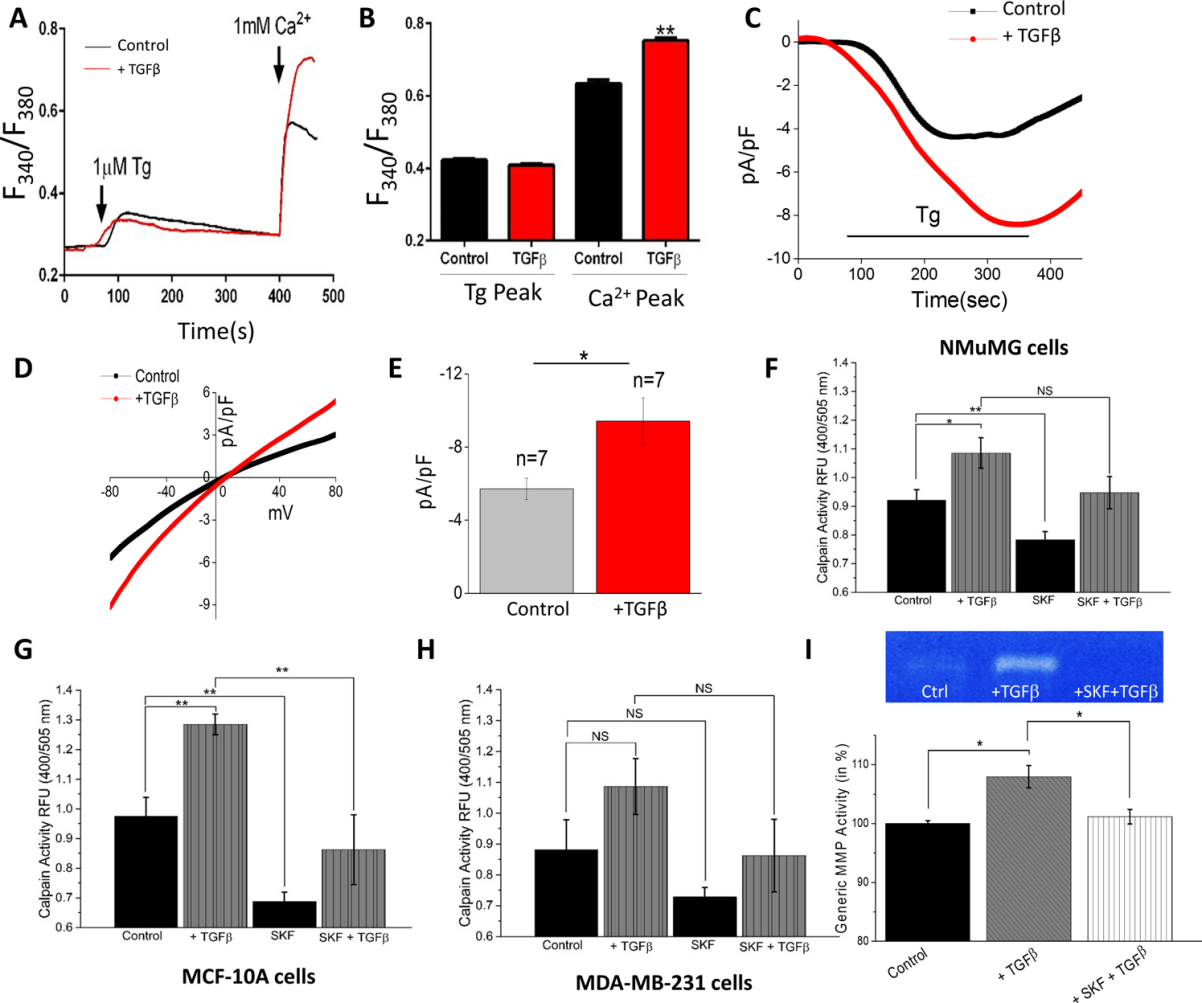
TGFβ Increases store-operated Ca^2+^ entry and [Ca^2+^]i and blocking Ca^2+^ influx reduces EMT calpain activity (**A**) Representative Fura-2 traces showing the transient increase in [Ca^2+^]i after addition of 1 mM calcium to NMuMG cells pretreated with TGFβ for 1 hour. (**B**) Bar diagram quantifies Fura-2 Tg and Ca^2+^ peaks. Each bar gives the mean ± SEM of 45–60 cells in three separate experiments. (**C**) Application 1 μM Tg in bath solution induced inward currents at −80mV in control and TGFβ treated cells. Respectively IV curves under these conditions are shown in (**D**) and quantitation (*n* = 7 recordings) of current intensity at −80 mV is shown in (**E**). (**F**–**H**) Calpain activity measured as RFU using calpain activity kit from Abcam, in NMuMG (**F**), MCF-10a (**G**), and MDA-MB-231 (H) cells after treatment with 10 ng/mL TGFβ and/or 10 μM SKF96365 for 6 hours. Each bar gives the mean ± SEM (*N* = 4 independent experiments). (**I**) Culture media of NMuMG cells were collected for generic MMP activity measurements after treatment with TGFβ and SKF for 12 hours and shown as bar graph. Gel image shows MMP activity in various conditions as labeled.

TGFβ has also been shown to regulate Ca^2+^ signaling [37–39], thus we investigated whether store depleted Ca^2+^ influx initiation (via the store-operated Ca^2+^ entry (SOCE) mechanism) is altered upon a short-term treatment of TGFβ. Importantly, in the absence of external Ca^2+^ (0 mM [Ca^2+^]_out_), addition of thapsigargin (Tg, a SERCA pump blocker that releases Ca^2+^ from the internal ER stores), did not show any statistical difference in control NMuMG cells or cells pretreated with TGFβ for 1 hour (Figure 2A, 2B). In contrast, addition of external Ca^2+^, which initiates Ca^2+^ entry, was significantly increased in TGFβ treated NMuMG cells (Figure 2A, 2B). These results suggest that TGFβ-induced activation of store-mediated Ca^2+^ entry could be important in promoting EMT. To establish the identity of the Ca^2+^ influx channel, electrophysiological recordings were performed on NMuMG cells treatment with TGFβ for 1 hour. Addition of Tg induced an inward Ca^2+^ current (recorded at −80 mV holding potential) which was non-selective in nature and the current reversed between 0 and −5 mV (Figure 2C). To delineate the properties of the channel, I/Vs curves were generated using a ramp protocol and the current density was evaluated at various membrane potentials (Figure 2D–2E). Importantly, the channel properties observed in NMuMG cells were similar to those previously observed with TRPC1 channels [40, 41], suggesting TRPC1 could contribute to the endogenous store-mediated Ca^2+^ entry channel in NMuMG cells. Importantly, cells treated with TGFβ showed a significant increase in SOCE currents without altering the current-voltage (I-V) relationship (Figure 2D, 2E). Similar results were obtained in MCF-10A cells, where TGFβ treatment increased store-mediated Ca^2+^ entry (data not shown). Overall, the data presented thus far suggests TGFβ-induced increase in SOCE-mediated Ca^2+^ entry could be involved in EMT.

### Blocking Ca^2+^ influx reduces EMT via inhibiting the calpain activity

Given the importance of Ca^2+^ homeostasis in regulating cell proteases, we next studied whether TGFβ induced increase in SOCE currents could activate Ca^2+^–dependent calpains, leading to the loss of E-cadherins observed above. Overall calpain activity was measured, which showed a significant increase in NMuMG and MCF-10A cells when pre-incubated with TGFβ for 6 hours (Figure 2F, 2G). MDA-MB-231 cells showed a slight increase in calpain activity when treated with TGFβ, however it was not significantly different from untreated cells (Figure 2H). To further establish that TGFβ induced calpain activation was dependent on Ca^2+^ influx, SKF96365 (SKF), a non-specific blocker of store-operated Ca^2+^ influx channels (including TRPC channels), was used. Importantly, all three cell lines demonstrated a reduction in calpain activity in SKF-treated cells and the TGFβ-induced increase in calpain activity observed in NMuMG and MCF-10A cells was attenuated by the addition of SKF (Figure 2F–2H). As expected, the increase in calpain activity seen with the addition of TGFβ was not significantly decreased by SKF in MDA-MB-231. These results indicate that TGFβ induced increase in SOCE currents could be responsible for activating calpains necessary for the disassembly of the extracellular matrix. To further our investigation of Ca^2+^ dependent ECM degrading proteases, we investigated whether TGFβ induced increase in SOCE currents could activate matrix metalloproteinases (MMPs). Total MMP activity of NMuMG cell culture media was measured using fluorometry, which showed a significant increase in activity when incubated with TGFβ for 12 hours (Figure 2I). When cells were treated with SKF alone, no change in activity level was observed as compared to control (data not shown). However, the increased activity seen with TGFβ treatment was attenuated by a blockage of Ca^2+^ entry by SKF. In addition TGFβ treatment increased MMP secretion as observed by gelatin zymography techniques, which was again attenuated upon blockage of Ca^2+^ entry by SKF (Figure 2I). Together these results indicate degradation of the ECM by TGFβ induced proteases could be regulated by SOCE.

### Blocking SOCE Ca^2+^ entry channels alters the effects of TGFβ

**Figure 3:**
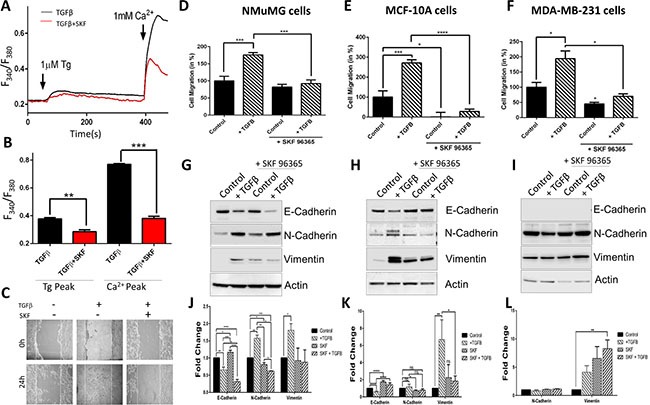
TGFβ induced Ca^2+^ entry, [Ca^2+^]i and migration is blocked by SKF96365 (**A**) Representative Fura-2 traces showing the transient increase in [Ca^2+^]i after addition of 1 mM calcium to NMuMG cells pretreated with 10 ng/mL TGFβ and TGFβ with 5 μM SKF96365 for 30 mins. (**B**) Bar diagram quantifies Fura-2 Tg and Ca^2+^ peaks. Each bar gives the mean ± SEM of 50–70 cells in at least three separate experiments. (**C**) Wound healing migration assay of NMuMG cells. Cells as indicating were treated for 24 hours, then confluent cells were treated with 10 mM Mitomycin C for 2 hours and then scratched indicated time 0. Transwell migration of NMuMG (**D**), MCF-10a (**E**) and MDA-MB-231 (**F**) cells treated with TGFβ (10 ng/mL) and/or 5 μM SKF96365 for 48 hours and migrated for 6 or 24 (MCF-10a) hours. Four random fields at 10x were counted indicating total cells migrated, shown as percent change. (**G**–**I**) Western blot showing E-cadherin, N-cadherin, and Vimentin protein levels normalized to actin with 40 ug total protein load after 48 hours in 10 ng/mL TGFβ and 5 μM SKF96365 treatment (along with quantification) in NMuMG (**G**, **J**), MCF-10a (**H**, **J**) and MDA-MB-231 (**I, L**).

Next we evaluated whether blocking store-mediated Ca^2+^ entry channels with SKF can alter the effects of TGFβ. NMuMG cells were treated with TGFβ and SKF and Ca^2+^ signaling, cell migration assays, and EMT factors were evaluated. The addition of Tg showed a significant reduction in internal Ca^2+^ release (first peak) in NMuMG cells treated with TGFβ and SKF for 30 minutes as compared to cells treated with TGFβ alone (Figure 3A, 3B). Importantly, a dramatic reduction in Ca^2+^ entry (in the presence of 1mM external Ca^2+^) was observed with the addition of SKF to TGFβ treatment. To determine whether Ca^2+^ is required for cell migration, wound healing assays were performed which showed that addition of TGFβ stimulated cell migration, whereas SKF treatment significantly reduced cell migration (Figure 3C). To further characterize morphological and migratory changes seen in TGFβ-induced EMT, NMuMG cells were pretreated with TGFβ and SKF and cellular motility was measured by transwell migration assay (Figure 3D). Treatment with SKF alone did not significantly alter migration of NMuMG cells, but with the addition of TGFβ there was a significant decrease in migration indicating Ca^2+^ dependency in TGFβ- induced EMT migration. Similarly, when MCF-10A cells were treated with a combination of TGFβ and SKF, there was a significant decrease in TGFβ-induced migration again indicating Ca^2+^ dependency in EMT migration (Figure 3E). In MDA-MB-231 cells, increased migration by TGFβ was also attenuated by SKF but to a lesser degree (Figure 3F). In the presence of SKF, basal migration (without TGFβ) was also significantly decreased in MCF-10A and MDA-MB-231. Importantly, our results show that all three cell lines when treated with both TGFβ and SKF, significantly decrease TGFβ-induced cell migration, suggesting Ca^2+^ is necessary for TGFβ-induced EMT migration (Figure 3D–3F).

Next we evaluated if SKF treatment also altered proteins essential for EMT. Analysis of E-cadherin showed that the addition of SKF to NMuMG cells increased E-cadherin expression and actually aided in the reduction of E-cadherin when combined with TGFβ (Figure 3G, 3J). Similar results were observed in N-cadherin and Vimentin where SKF increased their expression and the combination of TGFβ and SKF increased the effects of TGFβ on N-cadherin *per se*. However, SKF seems to attenuate the effects of TGFβ in Vimentin expression in NMuMG cells (Figure 3G, 3J). In MCF-10A cells, the addition of SKF again increased E-cadherin expression, however unlike the NMuMG cells, the addition of SKF to TGFβ treated cells attenuated the decrease in E-cadherin expression (Figure 3H, 3K). Similar results were observed in N-cadherin and Vimentin expression where SKF diminishes the effects of TGFβ. In MDA-MB-231 cells, the expression levels of N-cadherin and Vimentin remained the same in all three treatments (Figure 3I, 3L). Overall, TGFβ-induced migration in all three cell lines, but a change in EMT marker proteins was only observed in the epithelial NMuMG and MCF-10A cell lines. The presence of SKF significantly decreased TGFβ-induced cell migration in all three cell lines tested (Figure 3C–3F), however the TGFβ-induced alterations in EMT marker proteins was attenuated by SKF in MCF-10A and NMuMG cells only (Figure 3G, 3H). Together, this data suggests Ca^2+^ may be necessary for TGFβ-induced EMT migration and modifications of EMT factors in non-metastatic cells, whereas in metastatic cells migration is still dependent on Ca^2+^, but does not require adhesion proteins.

### siSTIM1 reduces the effect of TGFβ induced migration, calpain activity, and [Ca^2+^]_i_

**Figure 4:**
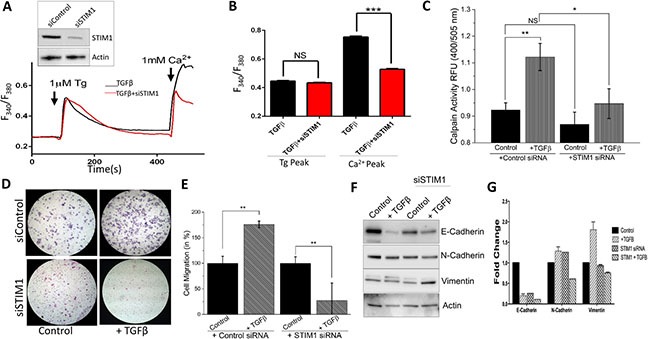
siSTIM1 reduces the effect of TGFβ induced migration, calpain activity, current, and [Ca^2+^]i (**A** inset) STIM1 was knocked down in NMuMG cells using siRNA with Western blot showing reduction. (**A**) Representative Fura-2 traces showing the transient increase in [Ca^2+^]i after addition of 1mM calcium to siSTIM1 cells and control cells with 10 ng/mL TGFβ pretreatment for 1 hour. (**B**) Bar diagram quantifies Fura-2 Tg and Ca^2+^ peaks. Each bar gives the mean ± SEM of 55 separate experiments. (**C**) Calpain activity measured as RFU using calpain activity kit from Abcam in siSTIM1 and control cells after treatment with 10 ng/mL TGFβ for 6 hours. Each bar gives the mean ± SEM (*N* = 4). (**D**) Transwell migration of siSTIM1 cells after treatment with 10 ng/mL TGFβ for 48 hours and migrated for 6 hours. Magnification at 10×. (**E**) Four random fields at 10× were counted indicating total cells migrated and shown as percent change. (**F**–**G**) Western blot of siSTIM1 cells showing E-cadherin, N-cadherin, and Vimentin protein levels normalized to actin with 40 ug of total protein load after 48 hours in 10 ng/mL TGFβ. Quantification displayed as fold change shown in (**G**).

To verify abnormal Ca^2+^ homeostasis is responsible for EMT, we silenced STIM1 as it has been shown as a critical activator of TRPC1 and ORAI1 channels and can induce Ca^2+^ entry through SOCE mechanisms. NMuMG cells treated with STIM1 siRNA showed a decrease in STIM1 levels without altering actin levels (Figure 4A, inset). In the absence of external Ca^2+^ (0 mM [Ca^2+^]_out_), measurements of intracellular Ca^2+^ changes by Tg treatment was not statistical different in NMuMG cells treated with TGFβ only as compared to cells treated with both TGFβ and siSTIM1 (Figure 4A, 4B). In contrast, the TGFβ-induced SOCE-mediated Ca^2+^ entry increase seen with the addition of external Ca^2+^ (1 mM) was attenuated by the expression of siSTIM1 in NMuMG cells (Figure 4A, 4B). Next we investigated whether the decrease in SOCE-mediated Ca^2+^ entry seen with siSTIM1 cells would affect the increase in TGFβ induced calpain activity. As expected, siSTIM1 cells diminished the increased calpain activity in TGFβ treated cells and no change in activity was observed in cells treated with siSTIM1 only (Figure 4C). These results indicate STIM1 may be partially responsible for the increased calpain activity induced by TGFβ. Additionally, siSTIM1 in TGFβ treated cells showed significantly reduced migration as compared to TGFβ only; again suggesting that STIM1 may be necessary for TGFβ induced EMT migration (Figure 4D). Moreover, siSTIM1 failed to show alterations in TGFβ–induced EMT marker protein levels (decrease in E-cadherin and subsequent increase in N-cadherin and Vimentin) (Figure 4F, 4G). Overall these results strongly suggest that TGFβ induced Ca^2+^ entry requires STIM1 and STIM1 is involved in regulating TGFβ-induced EMT.

### TRPC1 is required for TGFβ induced EMT

**Figure 5:**
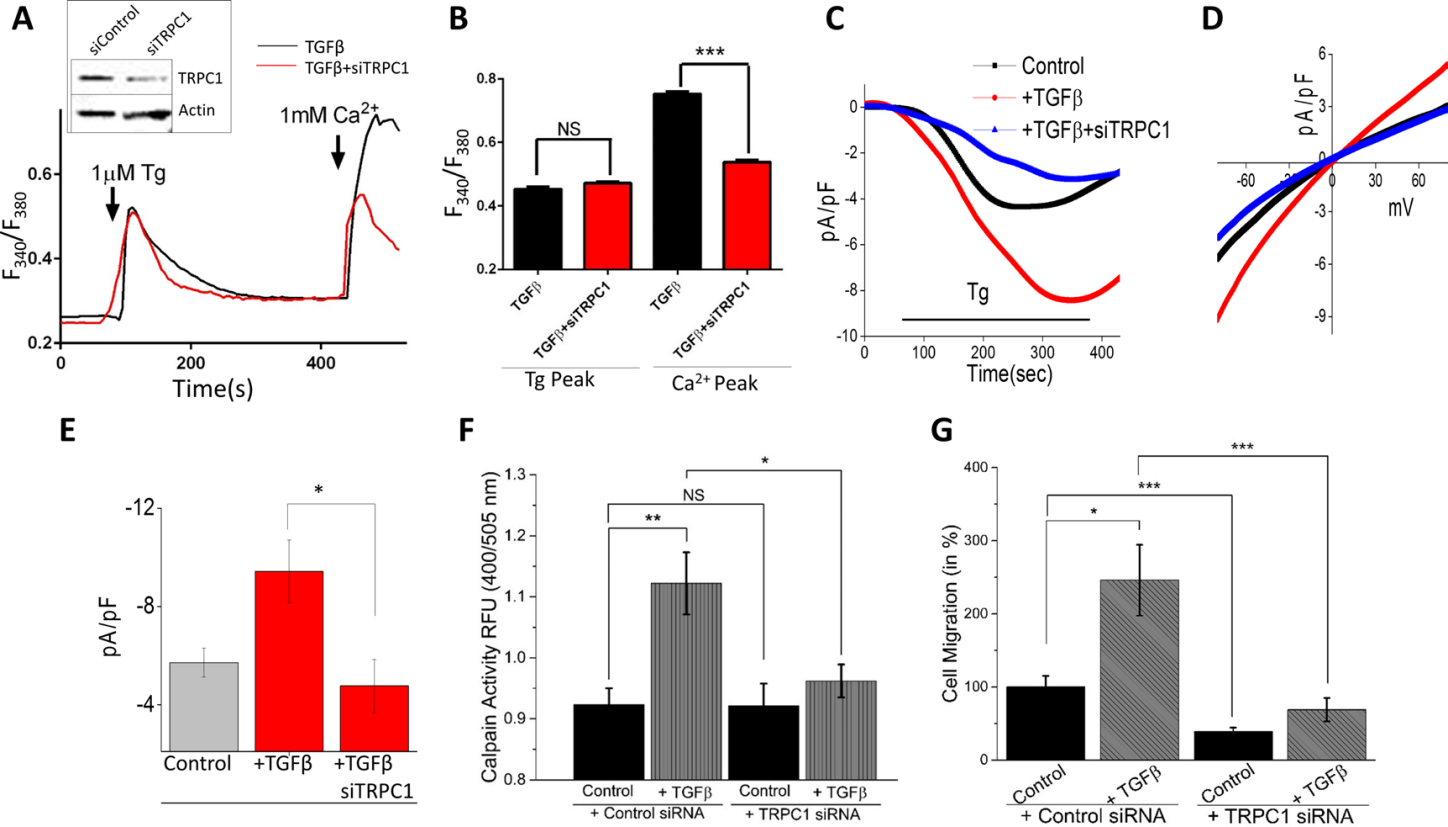
TRPC1 is involved in TGFβ induced EMT (**A** inset) TRPC1 was knocked down in NMuMG cells using siRNA with Western blot showing reduction. (**A**) Representative Fura-2 traces showing the transient increase in [Ca^2+^]i after addition of 1 mM calcium to siTRPC1 cells with TGFβ pretreatment for 1 hour. (**B**) Bar diagram quantifies Fura-2 Tg and Ca^2+^ peaks. Each bar gives the mean ± SEM of 40 separate experiments. (**C**) Application 1 μM Tg in bath solution induced inward currents at −80 mV in control,TGFβ treated and siTRPC1 cells. Respectively IV curves under these conditions are shown in (**D**) and quantitation (8–10 recordings) of current intensity at −80 mV is shown in (**E**). (**F**) Calpain activity measured as RFU using calpain activity kit from Abcam in siTRPC1 and control cells after treatment with 10 ng/mL TGFβ for 6 hours. Each bar gives the mean ± SEM (*N* = 4). (**G)** Transwell migration of siTRPC1 cells after treatment with 10 ng/mL TGFβ for 24 hours and migrated for 6 hours. Four random fields at 10× were counted indicating total cells migrated and shown as percent change.

Though no increase in TRPC1 expression levels was observed upon TGFβ treatment (data not shown), TRPC1 may be an integral part of EMT signaling. To study the importance of TRPC1 channels in TGFβ-induced EMT, we silenced TRPC1 in NMuMG cells using siRNA, which exhibited ~60% decrease in TRPC1 protein levels, without altering actin levels (Figure 5A, inset). Similar to siSTIM1 expressing cells, no change was observed after TGFβ treatment in intracellular Ca^2+^ changes by Tg treatment in siTRPC1 expressing NMuMG cells (Figure 5A, 5B). Conversely, the SOCE-mediated Ca^2+^ entry increase seen with TGFβ and the addition of external Ca^2+^ (1 mM) was attenuated by the expression of siTRPC1 in NMuMG cells (Figure 5A, 5B). To further establish TRPC1 is the major Ca^2+^ entry channel responsible for TGFβ-induced increase Ca^2+^ entry, electrophysiological recordings were performed. Store-depletion via Tg induced an inward Ca^2+^ current, which was significantly increased in TGFβ-treated cells (Figure 5C–5E). Importantly, TRPC1 silencing completely augmented TGFβ-induced Ca^2+^ entry increase in NMuMG cells without altering the current-voltage (I–V) relationship (Figure 5D, 5E). Similar results were also obtained in MCF-10A, where TGFβ treatment increased store-mediated Ca^2+^ entry (data not shown). Next we examined how a reduction in TGFβ-induced activation of SOCE-mediated Ca^2+^ by siTRPC1 affected calpain activity. As seen with siSTIM1 expressing cells, siTRPC1 expression abolished the increased calpain activity seen with TGFβ treatment without changing the basal calpain activity levels (Figure 5F). When observing the effects of siTRPC1 on cellular migration, siTRPC1 significantly reduced the increase in TGFβ-induced migration (Figure 5G). Together these results strongly suggest TRPC1 is the major Ca^2+^ entry channel that regulates TGFβ-induced EMT.

### TRPC1 overexpression increases the effect of TGFβ-induced EMT

**Figure 6:**
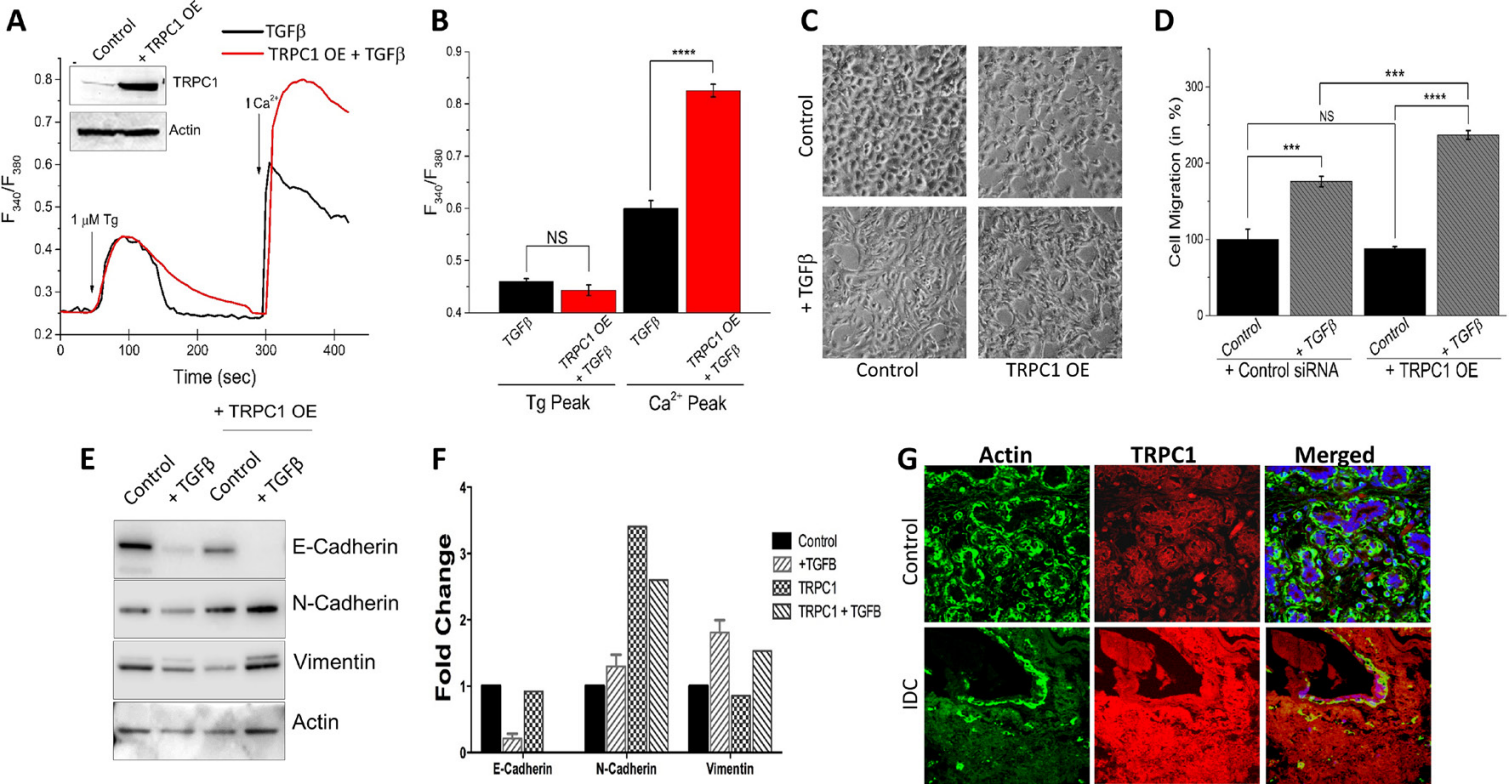
TRPC1 overexpression increases TGFβ induced EMT (**A** inset) TRPC1 expression vector showed a more than 2 fold increase in expression. (**A**) Representative Fura-2 traces showing the transient increase in [Ca^2+^]i after addition of 1 mM calcium to TRPC1 OE cells with TGFβ pretreatment for 1 hour. (**B**) Bar diagram quantifies Fura-2 Tg and Ca^2+^ peaks. Each bar gives the mean ± SEM of 40 separate experiments. (**C**) Transwell migration of TRPC1 overexpressed NMuMG cells with a 48 hour 10 ng/mL TGFβ treatment and 6 hour migration. Magnification at 10×. (**D**) Four random fields at 10× were counted indicating total cells migrated and shown as percent change. (**E**–**F**) Western blot of TRPC1 OE cells showing E-cadherin, N-cadherin, and Vimentin protein levels normalized to actin with 40 ug of total protein load after 48 hours in 10 ng/mL TGFβ. Quantification displayed as fold change shown in (**F**). Confocal images showing TRPC1 expression in age matched control and invasive ductal carcinoma (IDC) samples are shown in (**G**). Anti- actin or anti-TRPC1 antibody were used to label actin (as control) or TRPC1 protein followed by FITC (for actin) or rhodamine (for TRPC1) -conjugated secondary antibodies.

To confirm TRPC1 is important for EMT, we evaluated TGFβ-induced EMT migration in NMuMG cells that overexpress TRPC1. Overexpression of TRPC1 showed a more than two fold increase in TRPC1 protein levels, without altering actin levels (Figure 6A, inset). In the absence of external Ca^2+^ (0 mM [Ca^2+^]_out_), measurements of intracellular Ca^2+^ changes by Tg treatment was not statistical different in NMuMG cells treated with TGFβ only as compared to cells overexpressing TRPC1 and treated with TGFβ (Figure 6A, 6B). However, the SOCE-mediated Ca^2+^ entry was significantly increased with the addition of external Ca^2+^ in TGFβ treated cells overexpressing TRPC1 as compared to TGFβ only (Figure 6A, 6B). This further indicates the involvement of TRPC1 in TGFβ initiated SOCE-mediated Ca^2+^ entry. As can be seen in Figure 6C, NMuMG cells overexpressing TRPC1 have a slightly more elongated and spindle like phenotype as compared to control and these properties seem to be increased when treated with TGFβ. Consistent with the Ca^2+^ signaling data, TGFβ-induced cell migration was significantly increased upon TRPC1 overexpression when compared to both control and TGFβ treated cells (Figure 6D). Importantly, TRPC1 overexpression without TGFβ did not significantly alter basal migration (Figure 6D). TRPC1 overexpressed cells displayed no change in the expression of E-cadherin and Vimentin as compared with control (Figure 6E, 6F). However, the overexpression TRPC1 resulted in an increased expression of N-cadherin. When TRPC1 overexpressing cells were treated with TGFβ, the alterations of EMT marker proteins (decrease in E-cadherin and subsequent increase in N-cadherin or Vimentin) were amplified over TGFβ only (Figure 6E, 6F). We further studied the expression/localization of TRPC1 channels in human control (normal) and breast cancer tissue samples. Importantly, TRPC1 protein displayed a uniform plasma membrane expression along with some subplasma membrane staining of both acinar and ductal cells (Figure 6G). Conversely, a significant increase (~30%) in TRPC1 expression was observed in the invasive ductal carcinoma (IDC) tissue samples, whereas no increase in actin (used as control) staining was observed. Tissues without the TRPC1 antibody showed no staining (data not shown). Together these results strongly suggest that TRPC1 is the major Ca^2+^ entry channel that regulates TGFβ-induced EMT and its expression is increased in breast cancer tissues.

## DISCUSSION

Breast cancer is not only the most common cancer in women, but is also the leading cause of cancer death in women. Mortality from breast cancer is almost entirely due to invasion and metastasis of neoplastic cells from the primary tumors to distant organ sites [3–5]; therefore, identifying the mechanism(s) inducing cell migration is important to limit cancer induced death. The ubiquitous second messenger Ca^2+^ is one of the critical regulators of cell migration [26]. Cytosolic Ca^2+^ mediates several cellular signaling pathways that are related to cell proliferation, differentiation, and the increase in Ca^2+^ influx is essential for the migration of various cell types, including tumor cells [42]. Intracellular Ca^2+^ concentration is tightly regulated in normal cells, however abnormal activation of Ca^2+^ signaling machinery could lead to abnormal activation of proteins inducing metastasis. In non-excitable cells, one of the major sources of increases in intracellular Ca^2+^ is via the store-mediated Ca^2+^ entry (SOCE) mechanism. Ca^2+^ release from the intracellular ER stores facilitates Ca^2+^ influx from the extracellular space that together increases the cytosolic Ca^2+^ concentration [43]. Therefore, changes in the expression or function of Ca^2+^ influx channels or its regulators could affect the cytosolic Ca^2+^ homeostasis and its dependent cellular process, such as cell proliferation and EMT, which is observed in cancer metastasis. Thus, agents that block Ca^2+^channels could be potentially used as therapeutic options to inhibit metastasis.

Here we provide evidence that TRPC1 and STIM1 are essential for inducing EMT. Our data shows that loss of TRPC1 not only decreased Ca^2+^ influx, but also inhibited EMT. Agonist-mediated release of Ca^2+^ from the ER stores, initiates Ca^2+^ influx through the TRPCs or Orai channels. Although two different Ca^2+^ influx channels have been identified, their channel properties are quite distinct. Orai1 has been shown to exhibit an inward rectifying Ca^2+^ current that reverses at positive potential; whereas TRPC1 currents are non-selective in nature with a reverse potential at or around 0 mV. Our data shows that the endogenous properties of the Ca^2+^ channel was similar as that observed with TRPC1. Furthermore, TRPC1 silencing decreased SOCE currents and abolished the effect of TGFβ on Ca^2+^ influx and SOCE currents in these cells. Moreover increased expression of TRPC1 was observed in invasive ductal carcinoma, which is consistent with previous studies [44–47].

Depletion of Ca^2+^ from the ER also leads to the dissociation of Ca^2+^ from the EF hand domain of STIM1 proteins [48]. STIM1 has been identified as the molecular link between ER Ca^2+^ store depletion and SOCE activation. Oligomerization of STIM1, followed by STIM1 translocation to the ER plasma membrane junction is essential for the activation of TRPC1 channels that initiates Ca^2+^ influx. Importantly, loss of STIM1 in breast epithelial cells also leads to decreased Ca^2+^ influx and inhibited EMT. These results are consistent with previous findings where increase in STIM1 expression induced metastasis [49]. Moreover, our results showed that STIM1 silencing had a similar effect as was observed with TRPC1 silencing. These results are in contrast with a previous finding where STIM1 was found to be antimetastatic [50]. One possibility could be that STIM1 might have additional functions besides regulating Ca^2+^ signaling as it was initially identified as a stromal protein. In addition, TGFβ–induced migration could have multiple pathways and Ca^2+^, along with TRPC1, might be involved in some cases. Consistent with our results, increase in STIM1 expression has been observed in various cancer cells and has been associated with the risk of metastasis in cervical cancer [51, 46]. To understand the mechanism as to how TGFβ-mediated increase in Ca^2+^ entry modulates EMT, we focused our attention on proteins involved in cellular adhesion. Adhesion is critical for tumor metastasis and blocking Ca^2+^ influx could slow down the turnover of focal adhesion molecules, resulting in larger focal adhesions and consequently stronger adherence. Such strong adherence could thereby impede the fast migration of cells, including metastatic cancer cells and could be the possible mechanism by which TRPC1-STIM1 promotes EMT in breast cells.

E-cadherin is a subclass of the cadherin family that plays a major role in the maintenance of intercellular junctions in epithelial tissues. Proper function and expression of E-cadherin is necessary for a cell to be stable in the epithelial sheet and retain strong adhesion with the cellular matrix. EMT causes a reduction in E-cadherin leading to a loss of affinity for neighboring cells and the increased expression of N-cadherin and Vimentin seen in mesenchymal motility associated with human and mouse tumors. Interestingly, treatment with non-specific Ca^2+^ channel blocker SKF reduced TGFβ-induced migration, though E-cadherin expression varied between NMuMG and MCF-10A cells. To investigate these results, we studied proteins that are regulated by cytosolic Ca^2+^ levels. Importantly a decrease in calpain and MMP activity was observed when Ca^2+^ influx was inhibited by SKF, which could modulate proteins required for E-cadherin adherence. Consistent with this, TGFβ-induced increase in Ca^2+^ influx through TRPC1 and STIM1 was important for the activation of calpains. Furthermore, inhibition of TRPC1/STIM1-mediated Ca^2+^ influx inhibited TGFβ-induced increase in calpain activation and the loss of E-cadherins. In addition, cadherins are also Ca^2+^-dependent and are required for cell-cell adhesion [52, 53]. We suggest that calpain activity is involved in EMT and cleavage of E-cadherin via these calpains and MMP that could promote EMT.

Our results also showed that TGFβ-induced increases in N-cadherin and Vimentin were decreased upon inhibition of Ca^2+^ influx by SKF. Both of which are major cytoskeletal component of mesenchymal cells necessary for motility seen in EMT. Further, inhibition of TRPC1/STIM1-mediated Ca^2+^ influx also decreased N-cadherin and Vimentin expression when treated with TGFβ indicating a possible role in the ‘cadherin switch’. This is supported by previous work indicating the expression of both N-cadherin and vimentin proteins in breast cancer cells can be reduced by intracellular Ca^2+^ chelation and are partially regulated by Ca^2+^ channel TRPM7. Combined with our results, TRPC1/STIM1-mediated Ca^2+^ influx may not only be involved in E-cadherin reduction, but also in the expression of mesenchymal proteins necessary for motility, however more research is needed to confirm. Although other adhesion systems are also present, inactivation of these adhesion systems has little effect on cell-cell adhesion when cadherins are functioning, suggesting that cadherins play a major role in intercellular physical adhesion and the number of cadherin molecules expressed in a cell directly affects its adhesiveness and will regulate cell migration. Treatment of cell layers with anti-cadherin antibodies induces the dispersion of cells, and cadherins are thought to play a key role in breast cancers, where significant difference in E-cadherin and N-cadherin expression is correlated with grade of tumor differentiation. In summary our results suggest that TGFβ-induced EMT is dependent on Ca^2+^ entry via the TRPC1-STIM1 complex that leads to the activation of calpains and MMPs, which target proteins involved in cellular adherence and promote their migration. Thus, inhibition of TRPC1-STIM1 complex could be an attractive target for therapeutic intervention against breast cancer metastasis.

## MATERIALS AND METHODS

### Cell culture, siRNA transfection, and reagents

NMuMG, MDA-MB-231, and MCF-10A cells were obtained from ATCC and cultured in their respective medium along with various supplements as previously described [54, 55]. Cells were maintained at 37°C with 95% humidified air and 5% CO_2_ and were passaged as needed. Culture medium was changed twice weekly and cells were maintained in complete media, until reaching 90% confluence. siRNA targeting the coding sequence of human TRPC1 or STIM1 was obtained from Ambion Life technology and a FITC-conjugated non targeting siRNA (siControl) was used as control. Cells were transfected with individual siRNAs (200–300 pmol) using Lipofectamine RNAiMax in Opti-MEM medium as per supplier's instructions (Invitrogen). For migration assays, cells were treated with siRNA 24 hours prior to inducement with TGFβ and again 24 hours after inducement to sustain silencing through migration at time point 48 hours post TGFβ inducement.

### Migration assay

For transwell migration assays, monolayer cells were pretreated with TGFβ for 48 hours. Cells were trypsinized and 25,000 cells were resuspended in serum free media and placed in transwell inserts with 8 uM pore membranes (Corning). Lower wells contained DMEM + 10% FBS and cells were allowed to migrate for 6–24 hours. Cells on the inside of the transwell membrane were removed with a cotton swab and cells on the lower side of membrane were fixed and stained with hematoxylin. Four random fields at 10× were counted indicating total cells migrated.

### Wound assay

Cells were grown in a confluent monolayer in a 12 well plate. Cell were pretreated with TGFβ and/or SKF for 24 hours then treated with 10 mM Mitomycin C + serum free media for 2 hours to inhibit cell proliferation. At time 0, a wound was inflicted in the cell layer by scratching the plate with a sterile pipette tip. Plates were rinsed gently with media twice prior to incubation to remove non-adherent cells. Digital images of the wound were obtained at times 0 hours and 24 hours at 10× magnification.

### Immunoblotting

Cells were harvested and stored at −80°C. Crude lysates were prepared from NMuMG, MDA-MB-231, and MCF-10A cells as described previously [55, 56, 51]. Protein concentrations were determined, using the Bradford reagent (Bio-Rad), and 25–50 ug of proteins were resolved on NuPAGE Novex 4–12% Bis-Tris gels, transferred to PVDF membranes and probed with respective antibodies. Peroxidase conjugated respective secondary antibodies were used to label the proteins. The proteins were detected by enhanced chemiluminescence detection kit (SuperSignal West Pico; Pierce). Densitometric analysis was performed using imageJ analysis and results were corrected for protein loading by normalization for β-actin expression as described in [55].

### Immunohistochemistry

Paraffin embedded tissues from age-matched controls without cancer and infiltrating ductal carcinomas were obtained, and 10–12 μm thick cryosections were performed, permeabilized at room temperature with 0.1% TritonTM X-100 (Sigma-Aldrich) in phosphate buffered saline (pH 7.4), blocked (10% donkey serum and 5% bovine serum albumin in phosphate buffered saline), and probed overnight with respective primary antibodies in a hydrated chamber maintained at 4°C. Following incubation with primary antibodies, the slides were washed and reacted with fluorophore-conjugated respective secondary antibodies for 1 hour at room temperature. Thereafter, the slides were washed again and coverslip mounted using VECTASHIELD^®^ HardSet Mounting Media with DAPI (Vector Laboratories, Inc, Burlingame, CA, USA). Images were acquired at 63× magnifications using a confocal laser-scanning LSM 510 Meta Confocal Microscope (Carl Zeiss Microscopy, LLC, Thornwood, NY, USA). Total fluorescence from each section was quantified using the ImageJ program (NIH, Bethesda, MD).

### Calcium measurement

Cells were incubated with 2 uM Fura-2 (Molecular Probes) for 45 min, washed twice with Ca^2+^ free SES (Standard External Solution, include: 10 mM HEPES, 120 mM NaCl, 5.4 mM KCl, 1 mM MgCl_2_, 10 mM glucose, pH 7.4) buffer as described in [^57]^. For fluorescence measurements, the fluorescence intensity of Fura-2-loaded control cells was monitored with a CCD camera-based imaging system (Compix) mounted on an Olympus XL70 inverted microscope equipped with an Olympus 40× (1.3 NA) objective. A monochrometer dual wavelength enabled alternative excitation at 340 and 380 nm, whereas the emission fluorescence was monitored at 510 nm with an Orca Imaging camera (Hamamatsu, Japan). The images of multiple cells collected at each excitation wavelength were processed using the C imaging, PCI software (Compix Inc., Cranbery, PA), to provide ratios of Fura-2 fluorescence from excitation at 340 nm to that from excitation at 380 nm (F340/F380). Fluorescence traces shown represent [Ca^2+^]_i_ values that are a representative of results obtained in at least 3–4 individual experiments using 30–70 cells in each experiment.

### Electrophysiology

For patch clamp experiments, coverslips with cells were transferred to the recording chamber and perfused with an external Ringer's solution of the following composition (mM): NaCl, 145; CsCl, 5; MgCl2, 1; CaCl2, 1; Hepes, 10; Glucose, 10; pH 7.3 (NaOH). Whole cell currents were recorded using an Axopatch 200B (Axon Instruments, Inc.). The patch pipette had resistances between 3–5 M after filling with the standard intracellular solution that contained the following (mM): cesium methane sulfonate, 150; NaCl, 8; Hepes, 10; EGTA, 10; pH 7.2 (CsOH). With a holding potential 0mV, voltage ramps ranging from −100 mV to +100 mV and 100 ms duration were delivered at 2 s intervals after whole cell configuration was formed. Currents were recorded at 2 kHz and digitized at 5–8 kHz. pClamp 10.1 software was used for data acquisition and analysis. Basal leak were subtracted from the final currents and average currents are shown. All experiments were carried out under room temperature.

### Calpain activation assay

Two million cells were grown on 35-mm plates and the cells were treated with 10 ng/mL TGFβ for 24 hours. The cells were lysed and supernatant was used for the measurement of protein concentration. The cell lysate were diluted using extraction buffer and the activation was measured according to the manufacturer's instructions (Abcam, MA). The samples were analyzed at an excitation of 400 nm and emission at 505 nm using Multiskan spectrum fluorometer (Thermo labsystems) and the colorimetric reading was normalized with the respective total protein concentrations [58].

### MMP activity

Total generic MMP activity was determined by isolating culture media and measured using the SensoLyte 520 Generic MMP Assay Kit (Anaspec). Assay can detect simultaneously the activities of MMP-1, 2, 7, 8, 9, 12, 13 and 14. Conditioned media from M various conditions were analyzed for gelatin degradation by electrophoresis under non-reducing conditions on a SDS-polyacrylamide gel containing 1 mg/mL gelatin. The volume of media loaded per lane was standardized on the basis of the cell count. The MMP activity was indicated by white lysis zones which were revealed by staining with coomassie blue.

### Statistical analysis

Mean and standard error values were computed for all continuous variables and frequency distributions were calculated for all categorical variables. Statistical comparisons were made using Student's *t* test. All statistical tests were two-tailed with *p* < 0.05 considered to be significant unless otherwise indicated.
